# Supplementary figures and images for: Therapeutic Potential of Fingolimod and Dimethyl Fumarate in Non-Small Cell Lung Cancer Preclinical Models
Source: Int J Mol Sci. 2022 Jul 25;23(15):8192. doi: 10.3390/ijms23158192 (PMC9330228; doi:10.3390/ijms23158192)

Figure S1

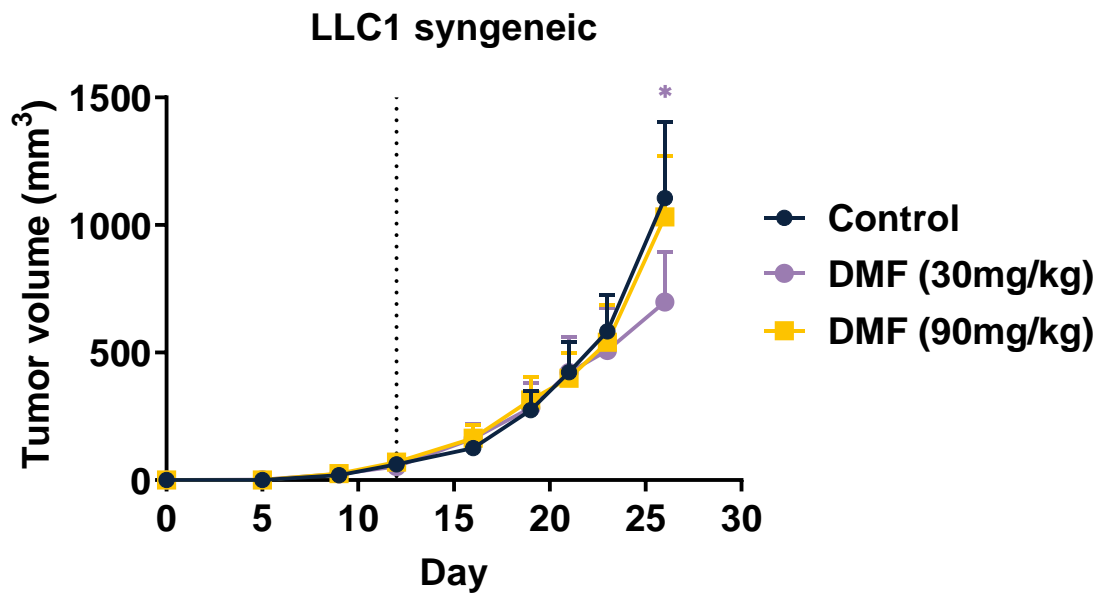

Supplement: Supplementary file 1 [file ijms-23-08192-s001.zip › Figure S1.pdf]

Figure S2

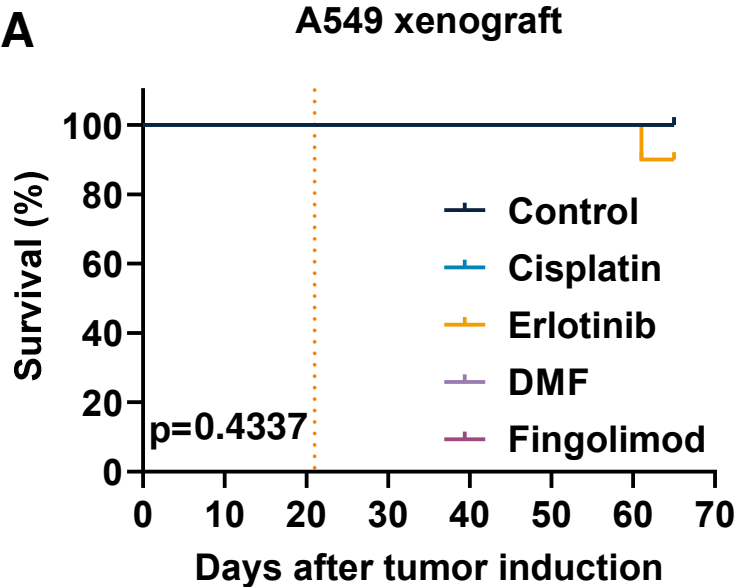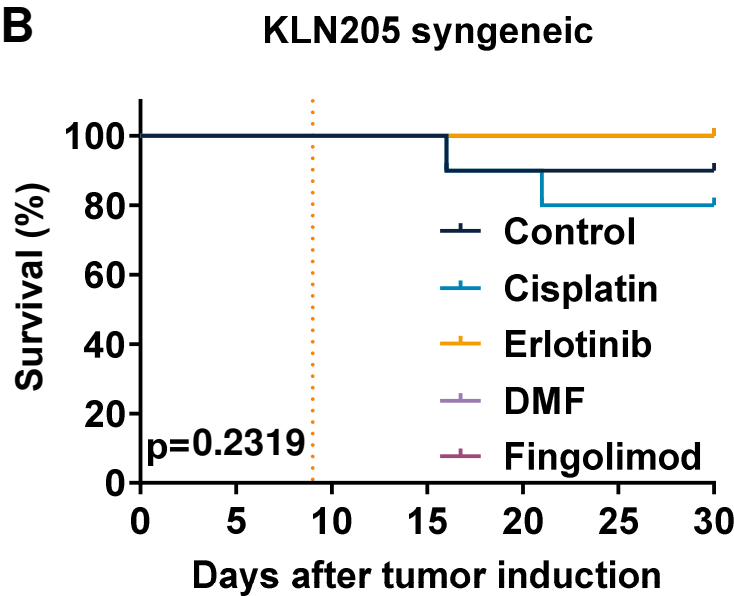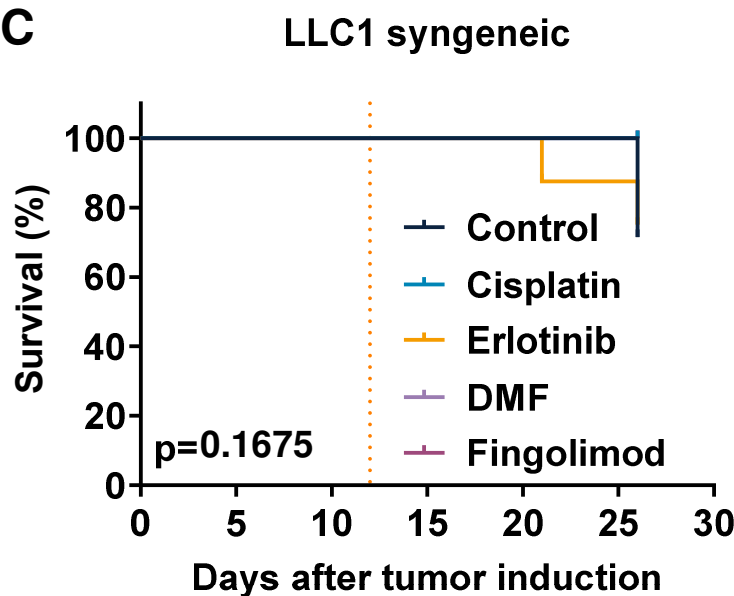

Supplement: Supplementary file 1 [file ijms-23-08192-s001.zip › Figure S2.pdf]

**Figure S3**

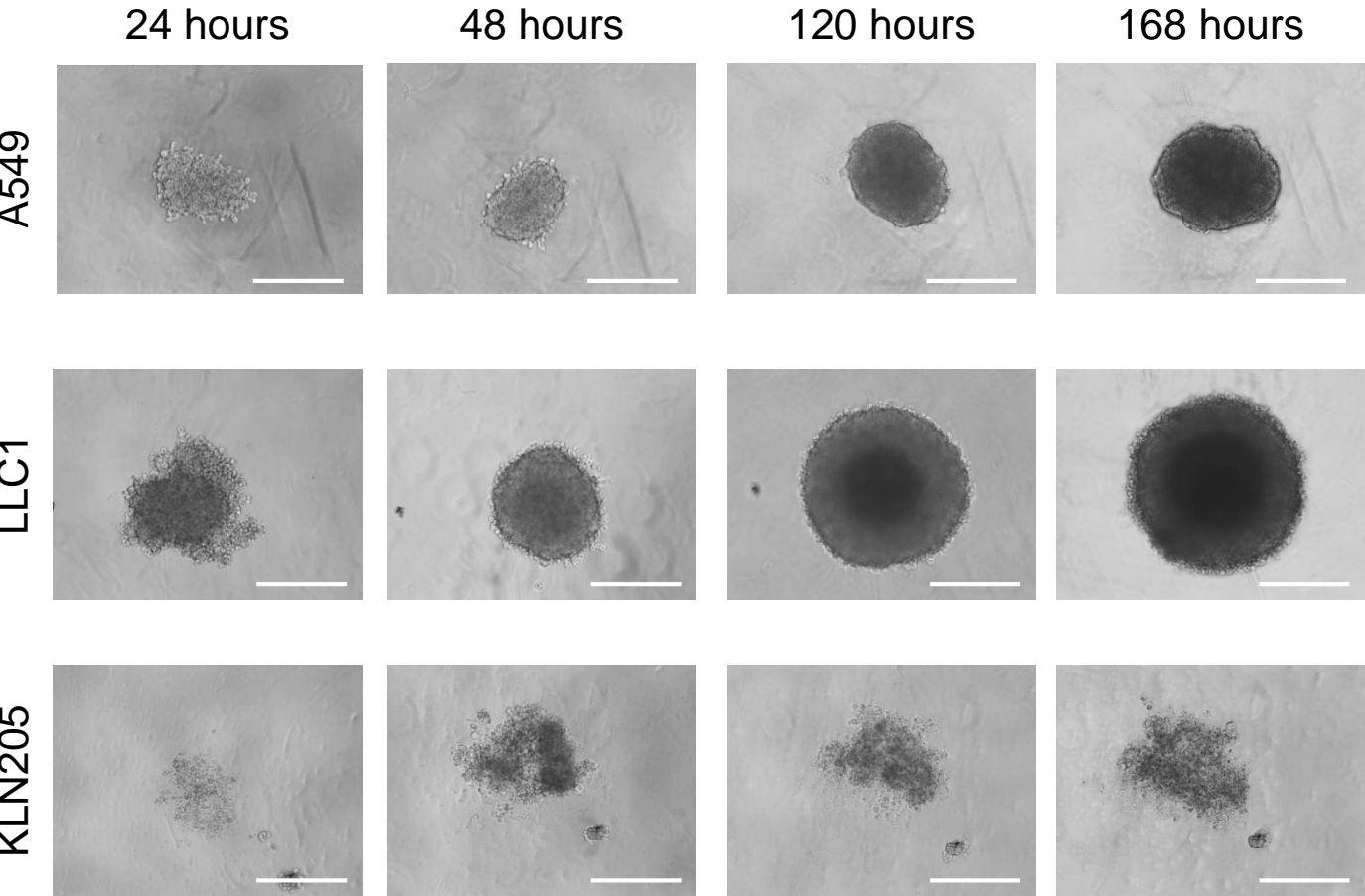

Supplement: Supplementary file 1 [file ijms-23-08192-s001.zip › Figure S3.pdf]

Figure S4

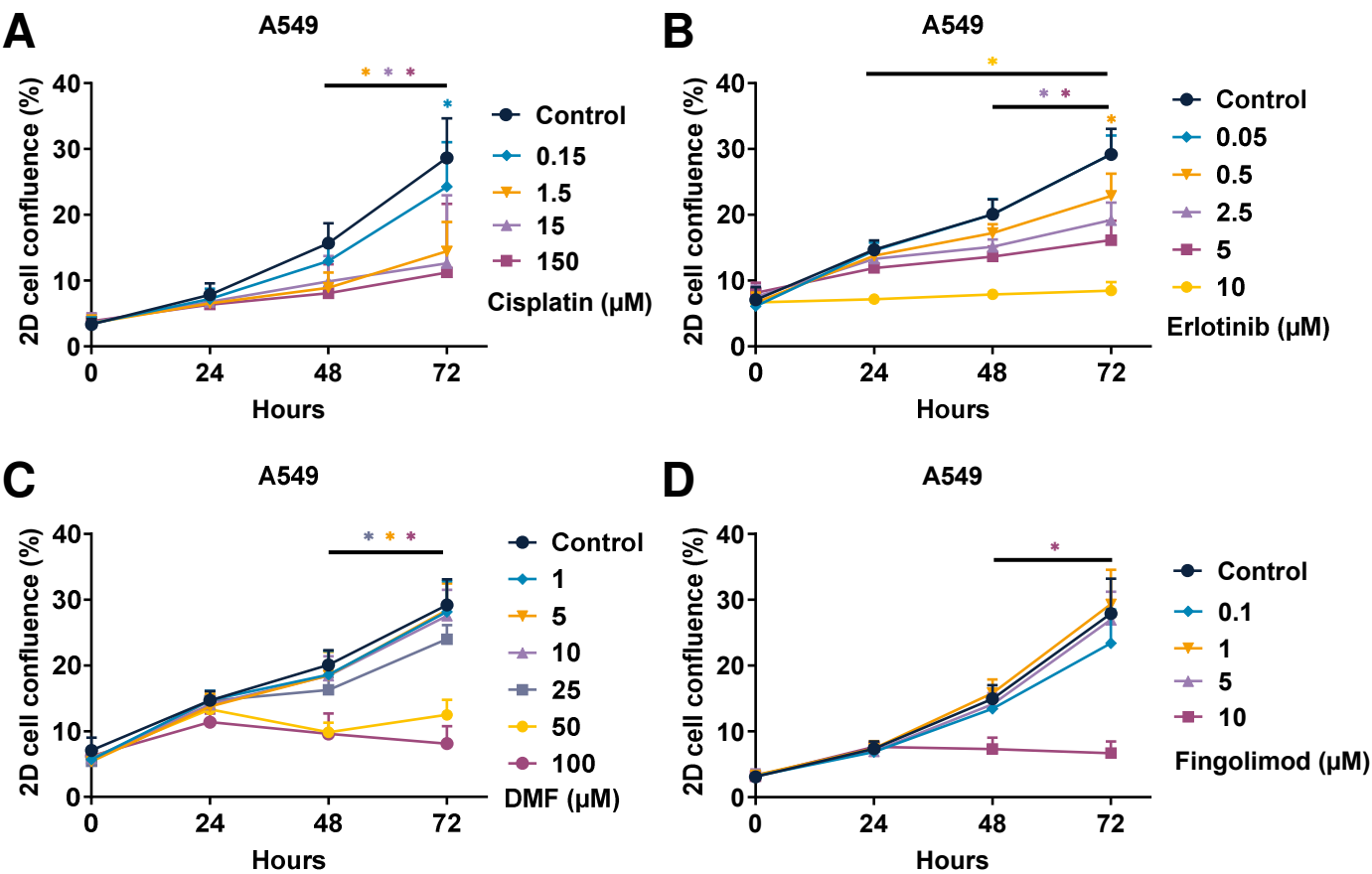

Supplement: Supplementary file 1 [file ijms-23-08192-s001.zip › Figure S4.pdf]
